# Emergency and Online Arbitration under the 2023 SCCA Rules: Efficiency, Due Process, and Enforceability in Saudi Arabia

**DOI:** 10.12688/f1000research.183752.1

**Published:** 2026-07-07

**Authors:** Hajed A. Alotaibi, Bandar A. Alyahya, Salem R. Alazizi

**Affiliations:** 1 Department of Sharia, College of Sharia and Law, Majmaah University, Al Majmaah, Riyadh Province, 11952, Saudi Arabia; 2 Department of Sharia Policy, Higher Institute of Judiciary, Imam Mohammad Ibn Saud Islamic University (IMSIU), Kingdom of Saudi Arabia, Imam Mohammad Ibn Saud Islamic University, Riyadh, Riyadh Province, Saudi Arabia; 3 Department of Law, College of Science and Theoretical Studies, Saudi Electronic University, Riyadh, Saudi Arabia

**Keywords:** Emergency Arbitration, Virtual Hearings, SCCA Rules 2023, Due Process, Enforceability

## Abstract

**Background:**

The 2023 Rules of the Saudi Center for Commercial Arbitration (SCCA) mark a significant shift in Saudi Arabia’s approach to procedural innovation, introducing an Emergency Arbitrator (EA) mechanism and comprehensive provisions for virtual and hybrid hearings. While these developments enhance procedural efficiency and align Saudi practice with global arbitration norms, they raise important questions regarding due process, enforceability, and compatibility with Sharia-based procedural principles.

**Methods:**

This article employs a doctrinal and comparative legal methodology, critically examining the legal foundations, procedural safeguards, and enforceability of the SCCA’s emergency and online arbitration provisions. The analysis draws on comparative institutional rules (ICC, SIAC, LCIA), Saudi arbitration and enforcement legislation, the New York Convention, and normative insights from Islamic jurisprudence.

**Results:**

The 2023 SCCA Rules largely succeed in balancing procedural fairness with operational flexibility. The EA mechanism provides rapid interim relief consistent with global standards, and the framework for virtual hearings integrates cybersecurity protocols and evidentiary safeguards. However, gaps remain regarding the legal characterization of EA decisions under the Saudi Enforcement Law, guidance on tribunal discretion, digital evidence standards, and cross-border enforceability. Sharia-based principles of harm prevention, fairness, and truth-seeking are broadly consistent with SCCA innovations but impose additional requirements on implementation.

**Conclusions:**

The article proposes a Hybrid Saudi Arbitration Model that integrates global best practices with Sharia-anchored notions of justice, accountability, and harm prevention. Strengthening due process protections, clarifying enforceability mechanisms, and standardizing virtual hearing protocols will be essential for positioning Saudi Arabia as a credible and competitive regional arbitration hub.

## 1. Introduction

Saudi Arabia’s arbitration landscape has undergone a profound transformation over the past decade, driven by institutional reforms, regulatory modernization, and a national strategy seeking to establish the Kingdom as a leading regional dispute-resolution hub. The 2023 Rules of the Saudi Center for Commercial Arbitration (SCCA) represent the most ambitious phase of this evolution, incorporating an Emergency Arbitrator (EA) mechanism and formalizing the use of virtual and hybrid hearings. These reforms reflect global developments in commercial arbitration particularly the expansion of urgent relief procedures and digital proceedings, yet they also present distinctive doctrinal and practical challenges within the Saudi legal environment.

The increasing reliance on emergency measures has reshaped the temporal dynamics of arbitration. The EA procedure is designed to provide urgent interim relief prior to tribunal constitution, thereby enhancing procedural efficiency and reducing the strategic misuse of delay. However, urgency compresses timelines and procedural protections, raising concerns about notice, equality of arms, and the risk of ex parte bias. These tensions mirror broader debates in procedural-justice scholarship, which caution that accelerated decision-making may undermine legitimacy if not balanced with fairness safeguards ( Pryce et al., 2025). Virtual hearings present complementary opportunities and risks: while enhancing access, reducing costs, and promoting continuity, they also introduce vulnerabilities related to authenticity of evidence, witness credibility, and cybersecurity.

Saudi Arabia’s distinctive normative framework adds additional layers of complexity. Recent research emphasizes the importance of harmonizing modern regulatory instruments with Sharia-based principles of justice, accountability, and harm prevention ( Alharthi & Alotaibi, 2026). In the Saudi context, arbitration procedures must therefore align not only with international standards but also with local expectations of procedural propriety and Islamic legitimacy. This dual mandate elevates the importance of doctrinal coherence and institutional competence, particularly in emerging areas such as digital evidence and online testimony.

The existing literature on Saudi arbitration acknowledges the Kingdom’s rapid institutional modernization but often stops short of critically evaluating how new procedural mechanisms reshape the balance between efficiency, fairness, and enforceability. Scholars have discussed governance harmonization, legal-Sharia integration, and institutional transformations in various sectors (e.g., Alotaibi, 2021), yet emergency and online arbitration remain underexamined in relation to enforceability tests applied by Saudi enforcement judges, cross-border recognition under the New York Convention, and the internal logic of Sharia-anchored due process.

This article addresses that gap by providing a doctrinally grounded, critically analytical assessment of the 2023 SCCA Rules. It evaluates whether the EA procedure and virtual hearings effectively balance efficiency with procedural fairness, and whether the resulting outcomes are enforceable under Saudi and international standards. It further assesses the extent to which SCCA reforms align with Islamic procedural principles relating to urgency, harm prevention, and equitable hearing.

The argument proceeds in eight sections. Section 2 situates the discussion within global arbitration developments. Section 3 provides a structural analysis of the 2023 SCCA Rules. Section 4 critically evaluates the tension between efficiency and procedural justice. Section 5 analyzes enforceability under Saudi and international law. Section 6 engages Sharia-based principles as a normative lens. Section 7 proposes a hybrid Saudi model reconciling innovation with doctrinal legitimacy. Section 8 concludes with broader implications for Saudi Arabia’s arbitral competitiveness.

## 2. Emergency and Online Arbitration in Global Context (Rewritten with Expanded In-Text Citations)

Emergency arbitration and virtual hearings have emerged as defining features of contemporary international arbitration, driven by institutional reforms and heightened demand for procedural adaptability. Understanding how these mechanisms function across leading arbitral systems is essential for assessing the innovations introduced by the 2023 SCCA Rules and for situating Saudi Arabia’s reforms within global legal trends.

### 2.1 Evolution of Emergency Arbitration Across Leading Institutions

Emergency arbitration was first formalized in the early 2010s by major arbitral institutions to provide immediate relief before constitution of the tribunal. The ICC Rules (2021) **, Article 29**, the SIAC Rules (2016) **, Schedule 1**, and the **SCC Rules (2017), Appendix II** all contain detailed EA procedures aimed at preventing irreparable harm. These frameworks share common elements: rapid appointment, abbreviated timelines, and broad interim powers.

**2.1.1 Accelerated Timelines and Compressed Procedure**

The ICC permits appointment of an emergency arbitrator within **24 hours** of receiving an application ( ICC Rules 2021, Art. 29(6)(b)). SIAC’s mechanism is even faster, requiring appointment within **one day** ( SIAC Rules 2016, Sch. 1, Rule 3). Scholarly commentary notes that this acceleration may compromise procedural fairness by limiting respondents’ ability to gather evidence or prepare submissions ( Born, 2021; Redfern & Hunter, 2020). These observations are consistent with broader procedural-justice research showing that compressed timelines can undermine perceptions of neutrality and participation ( Pryce, Alotaibi & Boateng, 2025).

**2.1.2 Scope of Powers and Interim Relief**

Emergency arbitrator mechanisms are generally designed to provide urgent protection before the constitution of the arbitral tribunal, thereby preserving the effectiveness of the arbitral process itself. Across major arbitral institutions, emergency arbitrators are empowered to grant a broad range of interim measures, including injunctions, asset preservation orders, security for costs, and directives intended to prevent imminent or irreparable harm. For example, Article 9B of the LCIA Rules (2020) authorizes emergency arbitrators to grant relief comparable to that available to a fully constituted tribunal. Nevertheless, such measures remain provisional by nature and are generally subject to review, modification, or confirmation by the subsequently constituted tribunal. This temporary character reflects a broader international consensus that emergency arbitration is intended to safeguard rights pending final adjudication rather than to determine the substantive merits of the dispute. Consequently, the relationship between interim relief and final enforceability continues to generate significant doctrinal debate within international arbitration scholarship ( Moses, 2020; Born, 2021).

**2.1.3 Enforceability Under the New York Convention**

The enforceability of emergency arbitrator decisions remains one of the most contested issues in contemporary arbitration law. The New York Convention of 1958 does not expressly address emergency arbitration orders within its definition of arbitral awards, resulting in divergent judicial approaches across jurisdictions. Some courts, particularly in Singapore and certain jurisdictions within the United States, have adopted a functional interpretation that permits enforcement where the emergency decision possesses sufficient adjudicative characteristics. Other jurisdictions continue to deny recognition on the basis that emergency measures are provisional rather than final determinations of rights and obligations. This fragmented landscape creates significant uncertainty for parties seeking urgent cross-border relief and highlights the importance of domestic legislative solutions capable of bridging the gap between arbitral necessity and judicial enforceability. The issue is particularly relevant to Saudi Arabia, where the interaction between emergency arbitrator decisions, the Arbitration Law, and the Enforcement Law remains an evolving area of legal development ( Marghitola, 2019; Scherer & Jensen, 2021).

### 2.2 Virtual and Online Hearings in International Arbitration

The COVID-19 pandemic accelerated the normalization of online proceedings, but the shift had begun earlier with the growth of digital evidence and remote-witness practice. Leading institutions now explicitly empower tribunals to order virtual hearings.

**2.2.1 Institutional Authorization of Online Hearings**

Virtual hearings have evolved from an exceptional procedural mechanism into a standard feature of international arbitration practice. Leading arbitral institutions increasingly grant tribunals broad authority to determine the format of hearings, including fully remote or hybrid proceedings. The ICC Rules, LCIA Rules, and UNCITRAL Arbitration Rules all reflect a growing recognition that technological tools can enhance procedural flexibility while reducing costs and delays. What began as a pragmatic response to logistical constraints has gradually developed into a permanent procedural option capable of accommodating increasingly international and geographically dispersed disputes. Contemporary scholarship suggests that the legitimacy of virtual hearings now depends less on their novelty and more on the procedural safeguards implemented to ensure fairness, participation, and evidentiary integrity ( Schweizer, 2021; Greenberg, 2023; UNCITRAL, 2013).

**2.2.2 Due-Process Concerns**

Academic and institutional analyses identify three principal risks:

1. **Witness integrity and credibility** — verifying identity and preventing off-camera coaching ( Schweizer, 2021).
2. **Digital inequality** — disparities in bandwidth, equipment, or cybersecurity resources affecting equality of arms ( Graziano, 2022).
3. **Data protection and cybersecurity** — heightened risk of interception or unauthorized access during remote hearings (ICC Commission Report, 2020).

Procedural-justice literature stresses that legitimacy depends not only on actual fairness but on the **perceived fairness** of procedures ( Pryce, Alotaibi & Boateng, 2025). Any discrepancy in technological access or audio-visual quality affects this perception.

**2.2.3 Consent, Discretion, and Hearing Format**

A key global debate concerns whether tribunals may impose virtual hearings without party consent.

• Under ICC and LCIA rules, tribunal discretion is broad.
• By contrast, some national laws—such as **German ZPO § 128a**—require explicit legal authorization.

Balancing tribunal discretion with party autonomy remains a core legitimacy issue.

### 2.3 Interplay Between Emergency Arbitration and Virtual Hearings

Emergency proceedings often take place online due to their urgency. This intersection compounds procedural vulnerabilities:

• shorter response windows,
• reduced opportunities for evidentiary challenge,
• higher dependency on digital submissions,
• increased risk of cybersecurity lapses under time pressure.

EA applications often include contested digital documents, screenshots, or voice recordings that require authentication—an issue highlighted in both comparative and Saudi academic writing on digital evidence and Sharia governance ( Alotaibi, 2021).

### 2.4 Implications for Saudi Arabia and the 2023 SCCA Rules

The global landscape suggests four critical lessons for Saudi Arabia:

A. **Procedural Efficiency Must Be Tempered with Sharia-Aligned Fairness**
  The Saudi **Arbitration Law (2012, amended 2023)** emphasizes party equality and due process (Art. 25). Compressed EA timelines challenge these principles unless offset by safeguards comparable to those in ICC or SIAC practice.
B. **Virtual Hearings Require Strong Digital Governance**
  The SCCA’s approach must integrate cybersecurity, authentication procedures, and access guarantees comparable to global institutions to maintain procedural legitimacy. This aligns with broader governance expectations found in Saudi scholarship emphasizing procedural integrity ( Alharthi & Alotaibi, 2025, 2026).
C. **Enforceability Requires Domestic Pathways**
  Under the **Saudi Enforcement Law (2012)** **, Arts. 11 and 34**, only “awards” may be enforced. Unless the SCCA clearly categorizes EA decisions as awards—or provides confirmation mechanisms—emergency relief may risk non-enforcement.
D. **Cultural and Normative Adaptation Is Essential**
  Islamic procedural principles such as *al-ʿadl* (fairness), *dar’ al-mafsadah* (prevention of harm), and *ḥisbah* (oversight) require contextualization when importing global arbitration practices. Saudi Arabia cannot rely solely on transplants from ICC or SIAC; it must adapt these practices to align with doctrinal expectations ( Alotaibi, 2021).

## 3. The 2023 SCCA Rules: A Structural and Doctrinal Overview

The 2023 Rules of the Saudi Center for Commercial Arbitration (SCCA) embody the most substantial reform in the institution’s history, reflecting global best practices while integrating procedural safeguards rooted in local legal culture. Two innovations stand out: the Emergency Arbitrator (EA) mechanism and the codification of virtual proceedings. This section examines the structure, procedural logic, and doctrinal underpinnings of these innovations, situating them within Saudi Arabia’s arbitration framework and Islamic-influenced procedural norms.

### 3.1 Overview of the 2023 Rules and Their Reform Trajectory

The revised SCCA Rules, effective May 2023, were drafted to align the Kingdom’s institutional arbitration framework with international standards observed in the ICC Rules (2021), LCIA Rules (2020), SIAC Rules (2016), and UNCITRAL model instruments. The Rules are intended to:

• increase procedural efficiency,
• enhance institutional credibility,
• support Vision 2030 objectives,
• strengthen enforceability of awards, and
• affirm Saudi Arabia’s position as a regional arbitration hub.

The reforms build upon the **Saudi Arbitration Law (2012, amended 2023)** and the **Enforcement Law (2012)**, both of which provide the statutory backbone for arbitral proceedings and award execution.

### 3.2 The Emergency Arbitrator Mechanism: Structure and Authority

**3.2.1 Triggering the EA Mechanism**

Articles 9–12 of the SCCA Rules establish the EA system. A party may apply **after filing a Notice of Arbitration but before tribunal constitution**, mirroring approaches under ICC Art. 29 and SIAC Sch. 1.

The SCCA must appoint an EA within *one day*, reflecting the urgency that characterizes comparable global frameworks. This mirrors SIAC’s ultra-expedited timelines and is quicker than the ICC’s 2-day standard ( ICC Rules 2021, Art. 29(6)).

**3.2.2 Scope of EA Powers**

The Rules grant the EA authority to issue **interim measures** similar to those available to fully constituted tribunals, including:

• preservation of assets,
• status quo orders,
• injunctions preventing irreparable harm,
• orders requiring security.

However, EA decisions are **interim and provisional**, requiring confirmation, modification, or revocation by the main tribunal—a safeguard aligned with global best practices.

**3.2.3 Safeguards: Notice, Participation, and Evidence**

The SCCA Rules mandate:

• timely notice to respondents,
• an opportunity to present submissions,
• transparent reasoning in EA decisions.

These obligations correspond to **Saudi Arbitration Law Art. 25**, which enshrines equality and full opportunity to present a case. They also reflect the procedural-justice concerns identified in comparative scholarship ( Pryce, Alotaibi & Boateng, 2025), ensuring that accelerated timelines do not compromise fairness.

### 3.3 Virtual Hearings and Digital Proceedings under the 2023 Rules

**3.3.1 Tribunal Authority to Order Virtual Hearings**

Article 32 explicitly authorizes **remote, hybrid, or fully virtual hearings**, entrusting tribunals with discretion to choose formats based on efficiency and fairness considerations. This aligns with ICC Art. 26, LCIA Art. 19, and UNCITRAL Art. 17(3).

**3.3.2 Digital Submissions, E-bundles, and Evidence**

The Rules encourage electronic filings, authenticated documentation, and platform-based case management. These provisions reflect international arbitration’s digital turn and respond to the Saudi judiciary’s broader transition toward e-justice infrastructure.

**3.3.3 Cybersecurity, Confidentiality, and Identity Verification**

The SCCA has published accompanying cybersecurity protocols addressing:

• encrypted communication channels,
• secure access to digital evidence,
• identity verification for witnesses,
• procedural remedies for breaches.

These measures respond to challenges identified in global scholarship regarding witness coaching risks, confidentiality breaches, and data interception ( Graziano, 2022).

### 3.4 Interaction with Saudi Arbitration and Enforcement Law

**3.4.1 Statutory Compatibility**

The 2023 SCCA Rules are designed to remain compatible with:

• Saudi Arbitration Law (2012/2023 amendments)
• Saudi Enforcement Law (2012)

The Arbitration Law grants parties autonomy to select institutional rules (Art. 4), and therefore SCCA procedures—including EA and virtual hearings—are fully recognized.

**3.4.2 Enforceability of EA Decisions and Online Awards**

A central doctrinal challenge concerns whether EA orders qualify as “awards” under **Enforcement Law Art. 11**. The Rules avoid direct conflict by allowing tribunals to **confirm EA measures** post-constitution, thereby producing a formally enforceable award.

Digital awards issued following virtual hearings are recognized under the Enforcement Law, provided they meet authentication and signature requirements. Saudi judicial practice, increasingly open to electronic evidence and digital processes, further supports enforceability ( Alharthi & Alotaibi, 2026).

### 3.5 Normative Foundations: Sharia Considerations in SCCA Reform

While the Rules are modeled on international practice, they also reflect Sharia-anchored procedural concepts:

• *Dar’ al-mafsadah* (prevention of harm) legitimizes urgent interim relief.
• *Al-ʿadl* (justice) requires equality of arms in expedited procedures.
• *Tahaqquq al-ḥaqq* (establishing the truth) supports flexibility in evidence presentation, including virtual formats.
• *Wujūb al-bayān* (duty of clarification) underpins reasoned awards and transparent emergency decisions.

These principles, articulated by contemporary scholars (e.g., Alotaibi, 2021 and Cizakça, 2019), reinforce the doctrinal legitimacy of SCCA innovations within Saudi Arabia’s legal identity.

### 3.6 Summary

The 2023 SCCA Rules represent a sophisticated institutional framework that:

• integrates global arbitration norms,
• responds to technological advances,
• aligns with Saudi statutory law, and
• remains consistent with the ethical and procedural expectations of Sharia.

The Rules create a principled foundation for emergency relief and virtual proceedings, but their effectiveness depends on how well they balance efficiency with fairness—a question examined in Section 4.

## 4. Efficiency and Procedural Justice: A Critical Evaluation

The 2023 SCCA Rules attempt to reconcile two competing imperatives in modern arbitration: the drive toward procedural efficiency and the obligation to preserve fairness, equality, and legitimacy. Emergency measures and virtual hearings exemplify this tension. This section critically evaluates the extent to which the SCCA framework succeeds in balancing these competing concerns, drawing from global standards, comparative jurisprudence, and empirical insights on procedural justice.

### 4.1 Efficiency as a Norm in Contemporary Arbitration

Global commercial arbitration has long prioritized procedural efficiency, often measured through reduced timelines, cost savings, speed of relief, and party convenience. Institutions such as the ICC, SIAC, LCIA, and SCC have systematically embedded expedited procedures, emergency mechanisms, and virtual hearings into their rules ( ICC Rules 2021, Arts. 29 & 30; SIAC Rules 2016, Sch. 1; LCIA Rules 2020, Art. 9). Efficiency is also a central element of Saudi Arabia’s broader judicial reforms under Vision 2030, reflected in initiatives that emphasize swift resolution and digital transformation.

The SCCA’s EA mechanism—requiring appointment within one day and decisions within 10–14 days—represents a strict adherence to global efficiency standards. Similarly, its permissive approach to online hearings vastly reduces scheduling delays and costs. In theory, the combination of both mechanisms positions SCCA as a competitive, modern arbitral institution.

However, efficiency is not normatively neutral. Accelerated processes can inadvertently marginalize due-process protections if tribunals lack adequate discretion or guidance. Scholarship warns that speed can “crowd out” fairness when procedural safeguards are insufficiently emphasized ( Born, 2021; Redfern & Hunter, 2020). This risk is heightened in legal systems where moral legitimacy and procedural propriety—such as those embedded in Sharia-influenced jurisprudence—are central to adjudicative authority.

### 4.2 The Due Process–Efficiency Paradox in Emergency Arbitration

Emergency arbitration is inherently asymmetrical: one party seeks urgent intervention; the other receives notice under compressed timelines. Comparative jurisprudence highlights several recurring risks:

**4.2.1 Limited Opportunity to Present a Case**

Even where notice is timely, respondents may face:

• insufficient time to assemble evidence,
• constraints on appointing counsel,
• inadequate preparation for cross-examination, and
• inability to rebut allegations in detail.

The SCCA Rules require that parties receive reasonable opportunity to present their case (Art. 18), echoing **Saudi Arbitration Law Art. 25**, which codifies equality of arms. Yet without explicit guidance on what constitutes “reasonable” within compressed EA timelines, tribunals risk inconsistent application.

**4.2.2 Procedural Disparities in Virtual Emergency Hearings**

When EA hearings are held virtually—as is common—the risks multiply:

• digital latency or poor connectivity may impair a party’s ability to argue effectively;
• identity verification procedures may be inadequate;
• urgent digital submissions may lack robust authentication;
• parties may be disadvantaged by unequal access to technology.

Empirical research on procedural justice confirms that parties evaluate procedural legitimacy based on fairness of voice, respectful treatment, and opportunity to participate ( Pryce, Alotaibi & Boateng, 2025). These perceptions may be compromised in virtual EA settings, where time pressures intersect with technological challenges.

### 4.3 Evidentiary Reliability and the Virtual Hearing Environment

Evidentiary issues are uniquely implicated in digital settings. Online proceedings alter conventional dynamics of:

• witness credibility assessments,
• control over the hearing environment,
• the risk of off-camera influence, and
• authenticity of electronic submissions.

The **ICC Commission Report on Information Technology (2020)** identifies witness coaching as a significant risk, recommending 360-degree cameras or room scans—techniques not yet fully standardized across institutions. Without similar protocols, SCCA tribunals may struggle to ensure evidentiary integrity.

Further, Saudi Arabia’s legal culture places strong emphasis on truth-seeking ( *tahaqquq al-ḥaqq*) and authenticity. A system that permits virtual testimony must therefore incorporate checks that honor these principles, especially where identity, intent, or contractual validity are in dispute.

### 4.4 Tribunal Discretion: Power Without Detailed Guidance?

While the SCCA Rules provide tribunals with substantial discretion—particularly regarding hearing format, timelines, and measures to ensure procedural fairness—they provide **limited detailed criteria** for exercising such discretion.

**4.4.1 Risks of Wide Discretion**

Wide discretion can lead to:

• inconsistent procedural outcomes,
• uncertainty about enforceability,
• perceptions of arbitral partiality,
• forum shopping between institutions.

Global scholarship argues that discretion without structured criteria increases unpredictability, which may deter users seeking reliable procedural frameworks ( Born, 2021; Moses, 2020).

**4.4.2 Need for Structured Due-Process Protocols**

Leading institutions such as the ICC and ICDR now publish **due-process protocols** or **checklists** guiding tribunals on:

• when to order virtual hearings,
• how to ensure witness integrity,
• how to balance efficiency with fairness,
• how to handle data-protection issues.

SCCA Rules contain cybersecurity guidelines but lack an explicit due-process protocol. Incorporating such guidance could enhance consistency and predictability.

### 4.5 Equality of Arms in Online Proceedings

The principle of equality is a fundamental requirement in both international arbitration and Saudi law. Procedural fairness under Sharia jurisprudence ( *al-ʿadl* ) also mandates that parties receive equivalent opportunities to argue and defend their cases.

Virtual hearings challenge this principle:

• Wealthier parties may have superior digital infrastructure.
• Parties operating from jurisdictions with weaker connectivity may be disadvantaged.
• Witnesses may have unequal access to secure environments.

To address these disparities, institutions such as LCIA mandate specific technological-adequacy standards ( LCIA Rules 2020, Art. 9A). The SCCA Rules mention “ensuring fairness” but could benefit from more explicit requirements.

### 4.6 Efficiency Gains Must Not Undermine Enforceability

Speedy EA awards and virtual-hearing awards are valuable only if enforceable. The **Saudi Enforcement Law (2012)** requires:

• authenticated awards,
• proper signatures,
• procedural compliance,
• absence of procedural unfairness.

International case law demonstrates that due-process irregularities—especially those arising from insufficient notice or technical disruptions—can result in non-recognition under **New York Convention Articles V(1)(b) and V(2)(b)**.

Thus, procedural shortcuts may jeopardize enforceability, undermining the very efficiency they aim to achieve.

### 4.7 Critical Assessment: Does the 2023 SCCA Framework Strike the Right Balance?

The analysis indicates that the SCCA has made substantial progress in aligning with global norms but faces several challenges:

**Strengths:**

• strong alignment with international standards,
• rapid EA mechanism,
• clear authority for virtual hearings,
• supporting digital infrastructure,
• Sharia-consistent procedural framing.

**Weaknesses/Risks:**

• limited guidance on tribunal discretion,
• potential inequality in digital capacity among parties,
• risk of perceived unfairness in compressed timelines,
• uncertain enforceability of EA decisions,
• lack of detailed evidentiary protocols for virtual environments.

Overall, while the SCCA framework generally balances efficiency and fairness, **its effectiveness ultimately depends on how tribunals operationalize discretion within the Saudi legal and cultural context**.

## 5. Enforceability Under Saudi Law and International Regimes

The enforceability of emergency measures and awards resulting from virtual proceedings is central to the overall effectiveness of the 2023 SCCA Rules. While efficiency and procedural flexibility are desirable, they are ultimately meaningful only if arbitral outcomes are capable of execution under Saudi law and recognized abroad. This section examines the enforceability of SCCA emergency decisions and virtual-hearing awards under:

(1) the Saudi Arbitration Law (2012, amended 2023),
(2) the Saudi Enforcement Law (2012), and
(3) the New York Convention (1958),

as well as the interplay of Sharia-based public-policy considerations.

### 5.1 Enforceability Under the Saudi Arbitration Law (2012/2023)

The Saudi Arbitration Law (SAL), modeled partly on the UNCITRAL Model Law, forms the statutory backbone of arbitral proceedings in the Kingdom. It emphasizes party autonomy, judicial non-interference, and procedural flexibility. Several provisions directly influence enforceability.

**5.1.1 Legal Recognition of Institutional Rules and EA Mechanisms**

Article 4 SAL Saudi Arbitration Law (2023) allows parties to adopt institutional rules including the SCCA Rules thereby validating emergency arbitration procedures even though SAL contains no explicit EA provisions. This statutory delegation ensures that SCCA emergency decisions are procedurally legitimate within Saudi law.

However, the absence of explicit statutory reference still creates interpretive ambiguity. Unlike Singapore, where the International Arbitration Act explicitly recognizes EA decisions, Saudi courts must infer EA validity from party autonomy. This could lead to inconsistent judicial approaches if enforcement judges question the legal nature of emergency decisions.

**5.1.2 Procedural Fairness Requirements**

Under Article 25 SAL, parties must be treated equally and given full opportunity to present their cases. Compressed EA timelines or technological barriers in virtual hearings may raise concerns if a party argues that insufficient notice, technical disruptions, or inability to participate undermined their procedural rights.

Saudi judges have historically emphasized fairness and opportunity to be heard—principles strongly rooted in Sharia procedural notions of *al-ʿadl* and *samāʿ al-ḥujaj.* Thus, EA decisions and virtual-hearing awards that deviate from these standards may face heightened scrutiny.

### 5.2 Enforceability Under the Saudi Enforcement Law (2012)

**5.2.1 Award Definition and EA Orders**

Under Article 11 of the Enforcement Law, only “final arbitral awards” may be enforced. Emergency arbitrator decisions—being interim and subject to modification—do not clearly fall within this category. To resolve this ambiguity, the SCCA Rules allow the main tribunal to **confirm** EA decisions, thereby transforming them into enforceable awards.

This confirmation mechanism mitigates risk but places a burden on parties seeking urgent enforcement. The lack of statutory clarity contrasts with more progressive regimes (e.g., Singapore), where EA orders are enforceable as awards in their own right.

**5.2.2 Digital Awards and Virtual Proceedings**

Saudi Arabia’s broader digital transformation has significantly influenced judicial attitudes toward electronic documents, digital signatures, and technology-assisted dispute resolution. As a result, awards rendered following virtual proceedings are increasingly capable of satisfying enforcement requirements, provided that appropriate authentication mechanisms are employed. Digital signatures, institutional verification systems, secure timestamps, and auditable electronic records collectively strengthen the reliability of electronically generated awards and support their recognition by enforcement authorities. Nevertheless, enforcement courts may still scrutinize the procedural integrity of the digital process itself, including the reliability of the technological platform used, the adequacy of cybersecurity protections, the effectiveness of witness identity verification procedures, and whether technical disruptions impaired the parties’ ability to participate on an equal basis. Where procedural records fail to demonstrate sufficient transparency or technological reliability, enforcement challenges may arise despite the substantive validity of the award ( Mason, 2021; Alharthi & Alotaibi, 2026).

### 5.3 The Role of Sharia Public Policy in Enforceability

Public policy remains one of the most significant mechanisms through which Saudi courts exercise supervisory control over arbitral awards. Although Saudi arbitration law generally reflects a pro-enforcement orientation, courts retain authority under Article 55 of the Arbitration Law to refuse recognition where enforcement would conflict with public policy principles. In the Saudi legal context, public policy is interpreted through the normative framework of Sharia, which prioritizes justice, fairness, transparency, and the prevention of harm. Consequently, the assessment of enforceability extends beyond technical procedural compliance and may encompass broader ethical considerations concerning the fairness of the arbitral process and the substantive implications of the resulting decision. This approach is broadly consistent with comparative international practice, where public policy exceptions continue to serve as a safeguard against procedural injustice while remaining subject to restrictive judicial interpretation ( Alharthi & Alotaibi, 2026; Gaillard & Savage, 1999).

**5.3.1 EA Decisions and Sharia Principles**

Because emergency arbitrator decisions are issued under compressed timelines and frequently involve urgent requests for interim protection, Saudi courts may subject them to closer scrutiny than final arbitral awards. Particular concerns may arise where relief is granted without adequate notice, where respondents are deprived of a meaningful opportunity to present their position, or where ex parte measures are adopted without compelling justification. From a Sharia perspective, such concerns engage fundamental principles of procedural justice and harm prevention, particularly the doctrine of *dar ʿal-mafsadah* and the broader requirement of equitable treatment between disputing parties. Accordingly, the enforceability of emergency measures is likely to depend not only on compliance with institutional rules but also on the demonstrable fairness and proportionality of the decision-making process itself ( Kamali, 2008; Alotaibi, 2021).

**5.3.2 Virtual Hearings and Witness Credibility**

Virtual hearings have generated renewed debate concerning witness credibility and the assessment of testimony in arbitral proceedings. Unlike traditional in-person hearings, remote proceedings may limit the tribunal’s ability to observe subtle behavioural cues and to verify the conditions under which testimony is delivered. Concerns frequently arise regarding the possibility of witness coaching, identity misrepresentation, unauthorized communications, or external influence occurring beyond the camera’s field of view. These concerns are particularly significant where witness testimony constitutes a central component of the evidentiary record.

From a Saudi legal perspective, such issues engage broader principles concerning evidentiary reliability and procedural fairness. Islamic jurisprudence has historically emphasized the importance of trustworthy testimony ( *shahādah*) and the integrity of the evidentiary process. While classical legal doctrine developed in a pre-digital environment, its underlying concern was not physical presence as an end in itself, but rather the ability to assess credibility and prevent deception. Consequently, virtual testimony is not inherently inconsistent with Sharia principles provided that appropriate safeguards are implemented to verify identity, preserve independence, and ensure the authenticity of evidence. Modern technological tools—including biometric verification, secure videoconferencing systems, digital audit trails, and recorded proceedings—may in some circumstances enhance rather than diminish evidentiary reliability. Nevertheless, tribunals must remain vigilant in balancing technological convenience with the fundamental objective of maintaining confidence in the truth-finding process ( Schweizer, 2021; Menon, 2022).

### 5.4 Enforceability Under the New York Convention (1958)

The enforceability of arbitral decisions remains the ultimate measure of arbitration’s practical effectiveness. While emergency arbitration and virtual proceedings offer significant procedural advantages, their legitimacy depends largely upon whether resulting decisions can be recognized and enforced both domestically and internationally. For Saudi Arabia, the New York Convention constitutes a critical legal framework governing the recognition and enforcement of foreign arbitral awards. Although the Convention has contributed significantly to the globalization of arbitration, emerging procedural innovations such as emergency arbitrator decisions and digitally conducted hearings continue to generate complex questions concerning finality, due process, and public policy. These issues are particularly relevant as arbitral institutions increasingly rely on technology-driven procedures that were largely unforeseen when the Convention was drafted in 1958 ( van den Berg, 2021).

**5.4.1 EA Decisions and Interim Measures**

Emergency arbitrator decisions continue to occupy an uncertain position within the global enforcement landscape. Because such decisions are intended to preserve rights pending the constitution of the arbitral tribunal, they are generally characterized as interim rather than final determinations. This distinction has led many courts to conclude that emergency orders fall outside the scope of the New York Convention, which was drafted primarily with final arbitral awards in mind. Nevertheless, several jurisdictions have adopted more flexible approaches by recognizing the practical necessity of emergency relief and by developing statutory mechanisms that facilitate enforcement. Singapore and Hong Kong are frequently cited as leading examples of jurisdictions that have explicitly incorporated emergency arbitrator decisions into their legislative frameworks. Saudi courts are likely to approach the issue cautiously, particularly where the emergency decision has not yet been confirmed by the subsequently constituted tribunal. Accordingly, the enforceability of emergency relief may depend less on the existence of the order itself and more on the procedural pathway through which it is integrated into the broader arbitral process ( Marghitola, 2019; Scherer & Jensen, 2021).

**5.4.2 Virtual Hearings and Due-Process Objections**

The increasing use of virtual hearings has introduced new dimensions to traditional due process analysis under Article V(1)(b) of the New York Convention. Parties resisting enforcement may argue that technological failures, unequal access to digital infrastructure, cybersecurity incidents, or logistical obstacles impaired their ability to present their case effectively. Such objections do not challenge the legitimacy of virtual hearings as a procedural format but rather focus on whether the specific circumstances of a given proceeding compromised procedural fairness. International practice suggests that courts are generally willing to uphold awards rendered through remote proceedings where tribunals have adopted reasonable safeguards to ensure participation, transparency, and equality of treatment. Consequently, the decisive issue is not whether a hearing occurred virtually, but whether the tribunal exercised its procedural discretion in a manner consistent with fundamental due process requirements ( Born, 2021; Greenberg, 2023).

### 5.5 International Enforcement: Comparative Risks and Opportunities

International enforcement introduces both opportunities and challenges for arbitral awards rendered under the 2023 SCCA Rules. On the one hand, Saudi Arabia’s modern arbitration framework, its adherence to the New York Convention, and the growing sophistication of the SCCA significantly enhance the credibility of Saudi-seated arbitration. On the other hand, the emergence of hybrid procedural models involving emergency relief, virtual hearings, and digitally authenticated evidence may generate divergent interpretations across jurisdictions. Some foreign courts may adopt expansive views regarding emergency arbitrator decisions and digital awards, while others may continue to apply more traditional concepts of finality and procedural regularity.

At the same time, technological developments create new opportunities to improve transparency, accessibility, and procedural efficiency. Properly authenticated digital awards, comprehensive electronic records, and secure hearing platforms may strengthen the evidentiary basis for enforcement by creating more detailed procedural documentation than is often available in conventional proceedings. The challenge therefore lies not in the technology itself, but in ensuring that technological innovation remains accompanied by robust procedural safeguards capable of satisfying diverse legal cultures and judicial expectations ( Paulsson, 2016; Queen Mary University of London, 2023).

### 5.6 Assessment: Toward a Coherent Enforceability Regime

The analysis suggests that the enforceability of emergency arbitrator decisions and awards rendered through virtual proceedings depends upon a combination of procedural rigor, technological reliability, and legal certainty. The 2023 SCCA Rules provide a strong institutional foundation by incorporating internationally recognized mechanisms while remaining compatible with Saudi arbitration legislation and broader Sharia-based principles of justice and fairness. Nevertheless, several areas would benefit from further clarification, particularly concerning the legal characterization of emergency arbitrator decisions, the standardization of virtual hearing protocols, and the development of detailed evidentiary guidelines for digital proceedings.

A coherent enforceability regime requires more than procedural efficiency alone. It also demands predictable standards governing notice, participation, evidence authentication, cybersecurity protection, and the exercise of tribunal discretion. Strengthening these areas would not only reduce enforcement risks but would also reinforce confidence among international users considering Saudi Arabia as a seat of arbitration. Ultimately, the long-term success of the SCCA framework will depend upon its ability to demonstrate that technological innovation can coexist with procedural fairness, doctrinal legitimacy, and international enforceability ( Moses, 2020; Born, 2021).

## 6. Sharia and Emergency/Online Proceedings: A Normative Inquiry

Any evaluation of the 2023 SCCA Rules must account not only for statutory and institutional compatibility but also for their coherence with Sharia-based procedural values. As Saudi Arabia’s arbitration framework is formally secular in operation yet substantively informed by Islamic legal principles particularly through public-policy review Sharia provides essential normative guidance Alotaibi (2020). This section explores how fundamental Islamic jurisprudential concepts align with, inform, or constrain emergency measures and virtual hearings.

### 6.1 Sharia’s Conception of Urgent Relief: Necessity, Harm Prevention, and Procedural Integrity

Emergency arbitration serves a preventive function: safeguarding parties from irreparable harm before a tribunal is formed. This rationale is consistent with several core Sharia doctrines:

**6.1.1 The Principle of Preventing Harm ( *Dar’ al-Mafsadah*)**

Classical jurists emphasize that preventing harm takes precedence over acquiring benefits. The maxim *dar’ al-mafsadah muqaddam ʿalā jalb al-ṣalāḥ* justifies urgent judicial intervention to avert irreversible injury.

This aligns directly with the SCCA Emergency Arbitrator’s mandate to act swiftly when delay would undermine justice.

**6.1.2 Necessity and Expediency ( *Ḥājah wa-Ḍarūrah*)**

Sharia recognizes that exceptional circumstances require procedural flexibility. Analogous doctrines exist in Islamic courts, where judges historically issued provisional orders ( *ḥajr*, *munāzaʿāt mustaʿjilah*) to prevent asset dissipation or preserve public order.

Emergency arbitration therefore fits within an established Islamic tradition of expedited judicial measures to prevent unjust outcomes.

**6.1.3 The Requirement of Procedural Balance**

Although urgency is justified, Sharia demands that expedited procedures never negate the right of the opposing party to be heard ( *samāʿal-ḥujaj*). Emergency arbitration’s compressed timelines must therefore preserve minimal procedural rights, including notice and opportunity to respond.

The SCCA’s safeguards—rapid but structured notice requirements—reflect this doctrinal balance.

### 6.2 Virtual Hearings and Islamic Evidentiary Doctrine

Virtual hearings raise important doctrinal questions concerning the relationship between technological mediation and evidentiary reliability. Classical Islamic procedural law attached considerable significance to the credibility of witnesses ( *ʿadālah*), the integrity of testimony ( *ḥifẓ al-shahādah*), and the conditions under which evidence was received and evaluated. At first glance, remote hearings may appear to challenge these assumptions because testimony is delivered through digital platforms rather than in a traditional adjudicative setting. However, a closer examination of Islamic legal theory suggests that the central concern has never been physical proximity as such, but rather the preservation of truth, authenticity, and procedural fairness.

Classical jurists accepted a variety of indirect evidentiary mechanisms, including written communications, delegated representation, documentary proof, and circumstantial evidence, provided that reliability could be established. Contemporary technological systems may perform similar functions by enabling secure identity verification, maintaining detailed procedural records, and preserving evidentiary integrity through digital authentication mechanisms. Consequently, the legitimacy of virtual hearings should be assessed according to their ability to achieve the objectives of reliable adjudication rather than by reference to physical presence alone. This functional approach is consistent with broader developments in Islamic legal reasoning that permit adaptation to changing circumstances where underlying legal objectives remain protected ( Kamali, 2019; Sait & Lim, 2021).

**6.2.1 The Legitimacy of Remote Testimony**

Classical fiqh does not categorically require the judge and witness to be physically co-present. Scholars recognized written testimony, delegated hearings, and indirect forms of proof under certain conditions. Modern Islamic-law scholarship similarly affirms that technological mediation is permissible if:

1. identity is verified,
2. witness independence is protected, and
3. truth-seeking is not undermined.

The SCCA’s digital-security protocols and identity-verification requirements support these conditions.

**6.2.2 Ensuring *ʿAdālah* in Virtual Environments**

Witness integrity ( *ʿadālah*) in Islamic law encompasses both moral character and reliability. Remote testimony risks undisclosed coaching or external influence, but these risks can be mitigated through:

• mandatory room scans,
• platform controls,
• tribunal oversight, and
• digital forensics.

By adopting measures similar to those recommended in global best-practice standards, SCCA tribunals can uphold the *ʿadālah* standard.

**6.2.3 Truth-Seeking ( *Tahaqquq al-Ḥaqq*) and Evidence Authentication**

Islamic law emphasizes the obligation to ascertain truth using the best available means. Digital evidence—screenshots, recordings, metadata—may strengthen documentary reliability if properly authenticated.

Saudi legal scholarship has emphasized that modern governance tools can support Sharia objectives by improving evidentiary accuracy ( Alotaibi, 2021). Thus, virtual hearings are doctrinally acceptable when they enhance or at least do not undermine evidentiary reliability.

### 6.3 Fairness, Equality, and Bias: Islamic Principles on Procedural Justice

Procedural fairness occupies a central position in both international arbitration and Islamic jurisprudence. Modern procedural justice research demonstrates that parties are more likely to accept legal outcomes when they perceive the process to be fair, transparent, and respectful, even when the outcome itself is unfavorable. Similar concerns are reflected in Islamic legal thought, where justice ( *al-ʿadl* ) and equality between disputing parties ( *musāwāt bayna al-khaṣmayn*) constitute foundational principles of adjudication.

The increasing reliance on digital technologies introduces new challenges to these principles. Differences in internet connectivity, technological literacy, access to secure digital infrastructure, and familiarity with virtual hearing platforms may create procedural asymmetries that are less visible than traditional forms of inequality. Accordingly, tribunals must actively ensure that technological advantages enjoyed by one party do not undermine the fairness of proceedings. This obligation extends beyond mere procedural formalities and reflects the broader Islamic requirement that adjudicative processes remain substantively just as well as procedurally correct. In this respect, the legitimacy of virtual arbitration depends not only on technological efficiency but also on the extent to which digital procedures preserve meaningful participation and equal opportunity to be heard ( Tyler, 2006; Pryce et al., 2025).

**6.3.1 Equality of Arms (Musāwāt baina al-Khaṣmān)**

The Qur’anic imperative of justice ( *iʿdilū huwa aqrab li-al-taqwā*) and juristic principles mandate equality between disputing parties. Differences in digital access or technical capacity could violate this principle unless tribunals intervene to level the procedural playing field.

**6.3.2 Non-Arbitrariness in Decision-Making**

Sharia prohibits arbitrary exercises of judicial authority ( *taḥakkum*). Emergency relief must therefore be based on clear legal standards, transparent reasoning, and demonstrable necessity.

The SCCA requirement for reasoned EA decisions aligns with this doctrinal obligation.

### 6.4 Sharia’s Perspective on Enforceability

**6.4.1 Binding Force of Arbitral Decisions**

Sharia recognizes arbitration ( *taḥkīm*) as a legitimate form of contractual dispute resolution and generally upholds arbitral decisions as binding upon the parties. The binding force of an arbitral award is contingent upon the existence of valid consent, the observance of procedural fairness, and the application of justice by the arbitrator throughout the proceedings. Equally important is the requirement that the award does not conflict with fundamental principles of public policy or established legal norms. These conditions reflect the broader Islamic commitment to fairness, accountability, and the protection of rights in dispute resolution. In this respect, the enforceability framework established under the 2023 SCCA Rules and the Saudi Arbitration Law is broadly consistent with classical Islamic understandings of arbitral legitimacy, as both systems seek to ensure that arbitration remains grounded in party autonomy while preserving procedural integrity and substantive justice.

**6.4.2 Public-Policy Objections Rooted in Sharia**

Although Saudi law generally adopts a pro-enforcement approach toward arbitral awards, courts retain the authority to refuse enforcement where an award conflicts with public policy principles derived from Sharia. Such objections may arise where an emergency arbitrator decision or an award rendered following virtual proceedings is found to undermine public morality, compromise procedural fairness, violate the integrity of the adjudicative process, or produce outcomes that are inconsistent with the Islamic principle of preventing harm. Consequently, the public policy exception operates as an important safeguard against procedural and substantive injustice, ensuring that the efficiency of arbitration does not override fundamental legal and ethical values. This approach closely resembles the operation of public policy exceptions under the New York Convention, while simultaneously maintaining fidelity to the doctrinal foundations of the Saudi legal system.

### 6.5 Normative Assessment: Are SCCA Innovations Sharia-Compatible?

The doctrinal analysis undertaken in this study indicates that the principal innovations introduced by the 2023 SCCA Rules are generally compatible with Islamic legal principles. Emergency arbitration reflects the longstanding Islamic concern with preventing imminent harm and preserving rights pending final determination, while virtual hearings may facilitate access to justice, reduce unnecessary burdens, and improve procedural efficiency. These objectives correspond closely with broader Sharia values relating to fairness, public interest, and effective dispute resolution.

At the same time, compatibility cannot be assessed solely at the level of abstract principles. The legitimacy of these mechanisms ultimately depends on implementation. Deficiencies in notice, inadequate identity verification procedures, weak cybersecurity protections, or excessive reliance on unstructured tribunal discretion may compromise the very values that emergency arbitration and virtual proceedings seek to advance. Accordingly, Sharia compatibility should be understood as a conditional rather than automatic conclusion, requiring continuous attention to procedural safeguards and institutional accountability. This perspective aligns with contemporary scholarship emphasizing that technological innovation must remain subordinate to the overarching objectives of justice and legal certainty ( Auda, 2021; Alotaibi, 2021).

## 7. Toward a Coherent Hybrid Saudi Model

The preceding analysis demonstrates that the 2023 SCCA Rules successfully integrate many features of contemporary international arbitration while remaining broadly consistent with Saudi legal principles. Nevertheless, the interaction between emergency arbitration, virtual hearings, enforceability requirements, and Sharia-based procedural values reveals the need for a more coherent conceptual framework capable of guiding future institutional development. Rather than viewing efficiency, due process, and religious legitimacy as competing objectives, the Saudi experience suggests that these values can be mutually reinforcing when embedded within a carefully designed procedural structure.

Accordingly, this study proposes a Hybrid Saudi Arbitration Model that combines three complementary dimensions: internationally recognized procedural standards, Sharia-based principles of justice and harm prevention, and domestic statutory requirements governing arbitration and enforcement. The objective is not to create a distinctively Saudi form of arbitration detached from international practice, but rather to demonstrate how global procedural norms may be adapted to local legal and cultural contexts without sacrificing efficiency, enforceability, or legitimacy. Similar approaches have emerged in comparative scholarship examining legal transplants and institutional adaptation within hybrid legal systems, where successful reform depends upon balancing international best practice with domestic normative foundations ( Berkowitz et al., 2003; Alasmari & Alotaibi, 2025).

## 8. Conclusion

The 2023 SCCA Rules signal a decisive shift in Saudi Arabia’s arbitral landscape, aligning the Kingdom with global innovations in emergency and online dispute resolution while navigating the doctrinal expectations of Sharia and the statutory frameworks of the Arbitration Law and Enforcement Law. This article has argued that although the new Rules successfully modernize procedural practice, their long-term legitimacy depends on how effectively they reconcile efficiency with procedural fairness, technological adaptation with evidentiary integrity, and international enforceability with Sharia-derived public-policy principles.

The comparative review in Section 2 showed that global arbitral institutions face similar tensions: urgent relief requires compressed timelines that may challenge due-process norms, and virtual hearings create opportunities for efficiency while introducing technological vulnerabilities. Section 3 demonstrated that the SCCA’s detailed structure—emergency powers, digital procedures, cybersecurity protocols—represents a sophisticated institutional response to these challenges. Yet Section 4 clarified that efficiency can undermine fairness unless tribunals implement structured safeguards, particularly in emergency contexts where notice, participation, and evidence review must remain meaningful despite rapid timelines.

Section 5 emphasized that enforceability—both domestic and international—hinges on procedural rigor. EA decisions risk non-recognition unless confirmed by the main tribunal, and remote-hearing awards must meet strict authenticity and fairness standards to survive judicial review under the Saudi Enforcement Law and the New York Convention. Section 6 showed that Sharia offers a rich set of principles— *dar’ al-mafsadah, al-ʿadl, ḥisbah, musāwāt al-khaṣmān*—that not only align with global norms but can enhance the legitimacy and coherence of modern arbitral practice.

Building on these insights, Section seven proposed a Hybrid Saudi Arbitration Model that integrates international best practices, Sharia-grounded procedural values, and enforceability-focused safeguards. Structured EA protocols, standardized virtual-hearing procedures, enhanced tribunal training, and digital-authentication standards together create a framework capable of addressing the unique demands of urgent and online proceedings in the Saudi context.

The article ultimately concludes that the SCCA’s reforms are directionally sound and doctrinally defensible but require continued institutional refinement. Strengthening due-process protections, clarifying enforceability mechanisms, and deepening the integration of technology with evidentiary standards will be essential to maintaining user confidence and judicial credibility. With these enhancements, Saudi Arabia is well positioned to establish itself as a leading arbitral jurisdiction one that combines procedural innovation with Sharia-anchored integrity and international enforceability.

## Data Availability

No datasets were generated or analyzed during this study. This article is a doctrinal and comparative legal analysis based entirely on published sources, legal texts, institutional rules, and academic literature cited in the references. No underlying datasets, numerical data, or empirical data are associated with this article.
