# Supplementary material for: Emergency and Online Arbitration under the 2023 SCCA Rules: Efficiency, Due Process, and Enforceability in Saudi Arabia
Source: F1000Res. 2026 Jul 7;15:1097. [Version 1] doi: 10.12688/f1000research.183752.1 (PMC13560060; doi:10.12688/f1000research.183752.1)
Supplement: Peer review history

# Reviewer report: Reviewer response for version 1

**DOI:** 10.5256/f1000research.202827.r501467

**Published:** 2026-08-03

**Authors:** Arief Budiono

**Affiliations:** 1 Universitas Muhammadiyah Surakarta (UMS), Kartasura District, Indonesia

This is an ambitious and well-organized doctrinal paper that takes on a genuinely underexplored niche: the intersection of Emergency Arbitrator (EA) mechanisms, virtual hearings, and Sharia-based procedural principles within Saudi Arabia's 2023 SCCA Rules. The eight-section structure builds logically toward a proposed "Hybrid Saudi Arbitration Model," and the tripartite framework used to assess enforceability — Saudi Arbitration Law, Saudi Enforcement Law, and the New York Convention — gives readers a clear doctrinal map. The paper's most original contribution is Section 6, where the author grounds virtual testimony in classical fiqh discussions of indirect evidence (written testimony, delegated representation) rather than treating physical presence as a fixed doctrinal requirement. Distinguishing "physical proximity as an end in itself" from "truth-preservation as the actual objective" is a genuinely non-obvious functional move, and it's the kind of reasoning that gives the paper real intellectual weight beyond a standard comparative-rules survey.

 The paper is also commendably honest about the SCCA framework's limitations — it doesn't oversell the reforms, explicitly flagging that EA decisions may not qualify as "awards" under Enforcement Law Art. 11 and that tribunal discretion lacks structured criteria.

 Authors could recheck, every citation to these recurring, less-familiar sources should be independently verified against the actual published text — confirm the title, journal, and that the cited proposition is what the source actually says — and any of that can't be verified should be replaced with established authority.

 Structurally, Section 7 — the Hybrid Saudi Model — is underdeveloped relative to how the abstract and title foreground it. Given that this is meant to be the paper's central proposal, one paragraph of assertion ("this study proposes...") is thin next to the five-plus pages of diagnostic analysis that precede it. Readers will expect concrete components (specific proposed rule or statutory amendments, e.g., how Enforcement Law Art. 11 might be redrafted to treat confirmed EA decisions as awards), a clearer account of how the model differs operationally from the current Rules, and ideally a comparative precedent showing a similar hybrid approach working elsewhere — Bahrain, DIFC-UAE, or Malaysia's Sharia-compliant arbitration regimes would all be natural points of reference. Relatedly, the enforceability claims in Section 5 rest almost entirely on comparative case law (Singapore, US) and general statements like "Saudi courts have historically emphasized fairness" without citing actual Board of Grievances (Diwan al-Mazalim) decisions or Ministry of Justice guidance — given that the paper's central claim is about enforceability under Saudi law specifically, this is a gap worth closing even with a small number of illustrative domestic cases.

 Overall, this is a strong draft after the major-revision stage, suitable for indexing. The doctrinal architecture and the Sharia-functionalist argument in Section 6 are genuinely indexable-quality contributions, and the paper's candor about the SCCA framework's gaps is a real strength rather than a weakness.

Is the case presented with sufficient detail to be useful for teaching or other practitioners?

Yes

Is the work clearly and accurately presented and does it cite the current literature?

Partly

If applicable, is the statistical analysis and its interpretation appropriate?

Yes

Are all the source data underlying the results available to ensure full reproducibility?

Yes

Are the conclusions drawn adequately supported by the results?

Yes

Is the background of the case’s history and progression described in sufficient detail?

Yes

Reviewer Expertise:

arbitration law

I confirm that I have read this submission and believe that I have an appropriate level of expertise to confirm that it is of an acceptable scientific standard.

# Reviewer report: Reviewer response for version 1

**DOI:** 10.5256/f1000research.202827.r501468

**Published:** 2026-07-27

**Authors:** Dwi Fidhayanti

**Affiliations:** 1 UIN Maulana Malik Ibrahim Malang, East Java, Indonesia

**Summary of the Article:**

 This manuscript thoroughly investigates the regulatory frameworks and socio-legal dynamics within the field. The authors clearly outline their research objectives, employ a well-structured methodology, and provide a detailed analysis of the core issues. By integrating relevant literature and empirical/theoretical insights, the study successfully highlights critical legal implications and offers valuable contributions to contemporary legal discourse.

**Evaluation:**

 The authors have meticulously addressed all comments from the previous review round. The theoretical foundation, methodological clarity, and argument flow have been significantly improved. The article now demonstrates high scientific rigor and clarity.

**Recommendation:**

**Approved / Accept for indexing.**

Is the case presented with sufficient detail to be useful for teaching or other practitioners?

Yes

Is the work clearly and accurately presented and does it cite the current literature?

Yes

If applicable, is the statistical analysis and its interpretation appropriate?

Yes

Are all the source data underlying the results available to ensure full reproducibility?

Yes

Are the conclusions drawn adequately supported by the results?

Yes

Is the background of the case’s history and progression described in sufficient detail?

Yes

Reviewer Expertise:

specializing in Islamic Economic Law (Hukum Ekonomi Syariah). Her research and expertise encompass three primary domains: Islamic Financial Law and Digital Economy, Socio-Legal Studies, Legal Curriculum Development and Academic Publishing.

I confirm that I have read this submission and believe that I have an appropriate level of expertise to confirm that it is of an acceptable scientific standard.
